# Biodistribution and function of extracellular miRNA-155 in mice

**DOI:** 10.1038/srep10721

**Published:** 2015-05-29

**Authors:** Shashi Bala, Timea Csak, Fatemeh Momen-Heravi, Dora Lippai, Karen Kodys, Donna Catalano, Abhishek Satishchandran, Victor Ambros, Gyongyi Szabo

**Affiliations:** 1 University of Massachusetts Medical School, Department of Medicine, Worcester, MA 01605; 2 University of Massachusetts Medical School, Department of Molecular Medicine, Worcester, MA 01605

## Abstract

Circulating miRNAs can be found in extracellular vesicles (EV) and could be involved in intercellular communication. Here, we report the biodistribution of EV associated miR-155 using miR-155 KO mouse model. Administration of exosomes loaded with synthetic miR-155 mimic into miR-155 KO mice resulted in a rapid accumulation and clearance of miR-155 in the plasma with subsequent distribution in the liver, adipose tissue, lung, muscle and kidney (highest to lowest, respectively). miR-155 expression was detected in isolated hepatocytes and liver mononuclear cells of recipient KO mice suggesting its cellular uptake. *In vitro*, exosome-mediated restoration of miR-155 in Kupffer cells from miR-155 deficient mice augmented their LPS-induced MCP1 mRNA increase. The systemic delivery of wild type plasma to miR-155 KO mice also resulted in a rapid accumulation of miR-155 in the circulation and distribution to the liver and adipose tissue. In summary, our results demonstrate tissue biodistribution and biologic function of EV-associated miR-155.

MicroRNAs (miRNAs) are a class of non-coding RNAs that regulate gene expression^1^. miRNAs are highly stable and can be detected in various human body fluids, including peripheral blood, plasma, and serum^2^,^3^,^4^. Circulating/extracellular miRNAs are present either in extracellular vesicles (EV) (including exosomes, microvesicles, and apoptotic bodies) or associated with non-membranous particles that include RNA inducing silencing complex (RISC) proteins (AGO proteins)^5^,^6^,^7^. Exosomes are of multivesicular bodies origin with a size range from 40–100 nm and express exosomal markers, including CD9, CD63, CD81, Alix, Flotillin-1 and Tsg101^8^. Microvesicles directly bud from the plasma membrane with a size range from 50–1000 nm and have no unique markers^8^. Currently consensus is still under development regarding the origin and nomenclature of exosomes and microvesicles.

Multiple lines of evidence indicate that circulating miRNAs have the potential to be distributed into organs via the circulation^9^,^10^,^11^. miRNAs are found to be stable in the circulation and this property makes them attractive for biomarker discovery. miRNAs are also receiving interest given their potential as therapeutic tools and targets in various diseases. Evidences suggest that extracellular vesicles (EV)/exosomes carry various cargos including nucleic acids (mRNA and non-coding RNA), proteins and lipids and serve as vehicles to transfer material between cells/organs^6^,^12^,^13^,^14^.

Exosomal meditated transfer of miRNA has been proposed to be a mode of intercellular communication^11^,^15^,^16^,^17^, and exosomes have been shown to play a role in inflammation and metastasis^13^,^18^,^19^,^20^. Moreover, exosomes have emerged as potential vehicles to deliver drugs, nucleic acids, and other molecules *in vivo*^21^,^22^,^23^,^24^. Studies have reported exosome-mediated delivery of siRNA into the brain and other organs^25^,^26^.

miR-155 is an inflammation associated miRNA that regulates inflammation and immune cell functions at multiple levels^27^,^28^. Previously, we showed induction of miR-155 in the plasma and liver after administration of inflammatory signals via TLR9 ligand (CpG) followed by TLR4 ligand (LPS) challenges^29^. Further, using the ExoQuick isolation method, we found that miR-155 was associated with extracellular vesicles/exosomes in the plasma^29^.

Though studies suggest that miRNAs are present in cell free forms, it is not clear if extracellular miRNAs in blood/plasma are present in physiologically relevant amounts to exert cell-to-cell signaling effects^30^. Little is known about the half-life and biodistribution of circulating miRNA. Using a miR-155 knock-out (KO) mouse transfer model system, here, we report the half-life and biodistribution of miR-155 mimic loaded in exosomes (exo-miR-155 mimic) and wild type plasma enriched in miR-155 (WT plasma). The latter represents both EVs and non-EV associated miR-155. Our results indicate a rapid accumulation of exosome-loaded miR-155 mimic (exo-miR-155 mimic) in the plasma of recipient miR-155 KO mice with subsequent distribution to the liver, adipose tissue, lung, muscle, and kidney (highest to lowest, respectively). At the cellular level, miR-155 was detected in hepatocytes and liver mononuclear cells (MNCs; monocytes, lymphocytes and dendritic cells) of recipient miR-155 KO mice *in vivo*, suggesting successful uptake of exo-miR-155 mimic by various populations of liver cells. Functionally, exo-miR-155 mimic was able to induce MCP1 in Kupffer cells isolated from miR-155 KO mice in an *in vitro* co-culture system.

To mimic the half-life and distribution of miRNA naturally present in the plasma, we administered WT plasma (donor) to miR-155 KO (recipient) mice. Our results indicate detection of donor miR-155 in plasma of recipient miR-155 KO mice and distribution to the liver and adipose tissue.

## Results

### Biodistribution of exosome-loaded miRNA-155 mimic in miRNA-155 deficient mice

**Figure 1:**
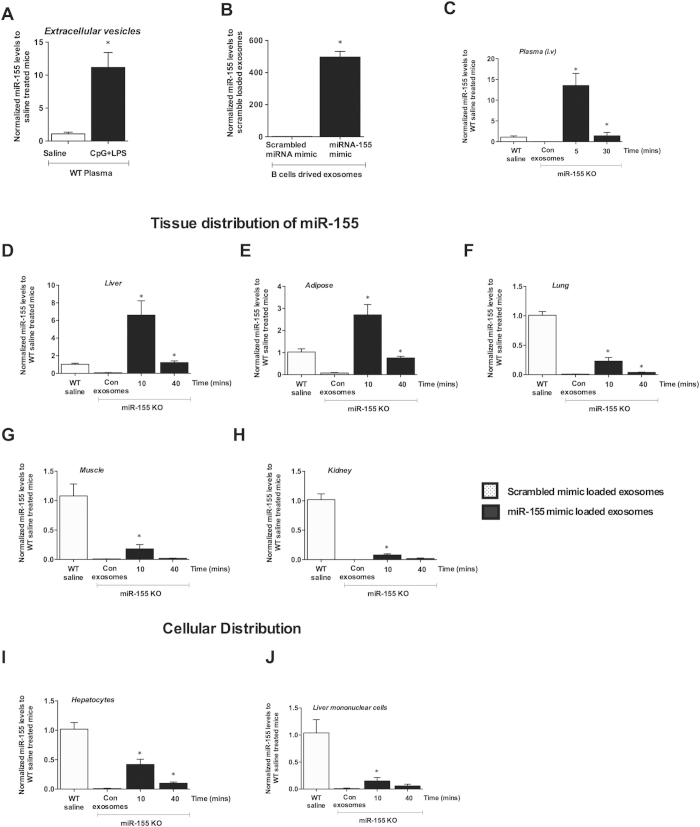
Biodistribution of exosome loaded miRNA-155 mimic in miRNA-155 KO mice. C57Bl/6 wild type female mice were either injected with saline or 2.5 mg/kg CpG DNA (i.p.) once a day for three days and on day 4 CpG treated mice received 0.5 mg/kg LPS for 3h and blood was isolated. Extracellular vesicles were isolated from plasma using filtration and ExoQuick and miR-155 levels were determined (**A**) B cells were treated with CD40 and IL-4 and exosomes were isolated using CD63 magnetic beads as described in the methods. Exosomes were electroporated with either scrambled mimic or miRNA-155 mimic and miR-155 levels were determined after RNase A treatment. miR-155 levels were normalized to scrambled mimic loaded exosomes (**B**) 100 ul exosomes (loaded with scrambled mimic or miR-155 mimic) were injected (i.v) into miR-155 KO mice for indicated times and mice were perfused as described in the methods. miR-155 levels in the plasma (**C**) liver (**D**) adipose tissue (**E**) lung (**F**) muscle (**G**) kidney (**H**) isolated hepatocytes (**I**) and mononuclear cells (**J**) was determined using real time qPCR. miR-155 levels were normalized to wild type saline treated mice. * indicates p < 0.05 versus scrambled mimic exosome treated KO mice. Synthetic spiked cel-miR-39 (**A**–**C**) or SnoRNA202 (**D**–**J**) was used to normalize the technical variations between the samples. Statistical analysis was performed using non-parametric Mann-Whitney test.

Circulating miRNAs are associated with either proteins (Ago2)^3^,^5^ or extracellular vesicles including exosomes/microvesicles^31^. Using filtration and ExoQuick isolation method we found that miR-155 was associated with extracellular vesicles in the plasma after CpG+LPS treatment in wild type mice (Fig. 1A). However, little is known about the half-life and biodistribution of exosome/microvesicles (EV) associated miRNAs (EV-miRNAs) *in vivo*. Here, we established an exosome-based miR-155 mimic delivery system to study the biodistribution of EVs associated miRNA-155 using a miR-155 KO mouse model.

To deliver miR-155 mimic, CD63 enriched exosomes were isolated from murine B cells using immune-magnetic bead selection as described in the methods^32^. CD63-enriched exosomes have an average size of 84 nm as determined by Nanosight (Suppl. Fig. 1A) and showed a typical concave/mushroom like morphology under transmission electron microscopy (Suppl. Fig. 1B)^22^. To rule out the possibility that electroporation may adversely affect exosomes structural stability, exosomes before and after electroporation were visualized by the electron microscopy. No significant differences in the structure of exosomes were found before and after electroporation (Suppl. Fig. 1B).

Exosomes isolated from B cells were enriched in CD63 and CD81 and free of any cellular protein contamination (Suppl. Fig. 1C). GRP78, an endoplasmic reticulum protein was present in the cells but absent in exosomes preparation (Suppl. Fig. 1C). Exosomes derived from B cells provided a suitable system for miR-155 mimic delivery since we found very low levels of miR-155 at the baseline^22^. Compared to exosomes isolated from naïve cells, miR-155 expression was decreased in exosomes derived from treated B cells (IL-4 and CD40) (Suppl. Fig. 1D)^22^.

The electroporation of miR-155 mimic (mature form of miR-155) and not miRNA mimic negative control #1 (scrambled mimic) resulted in a significant increase of miR-155 in exosomes (Fig. 1B). Further, no induction of pro-inflammatory cytokines (TNFα, MCP1 and IL-1β) both at the mRNA (Suppl. Fig. 1E) and protein levels (Suppl. Fig. 1F) were found in liver mononuclear cells (MNCs) isolated from WT mice treated with scrambled miRNA mimic loaded exosomes. To reduce the possibility of miRNA being outside of exosomes after electroporation, RNAse A treatment was performed. Real-time PCR data indicates that the amount of miR-155 was reduced in the re-isolated exosomes after RNAse A treatment compared to RNAse A naïve exosomes, suggesting degradation of free floating miR-155 mimic (Suppl. Fig. 1G). Based on these observations, the miR-155 mimic loaded exosomes (exo-miR-155 mimic) represented a suitable vehicle for testing the *in vivo* biodistribution of extracellular miR-155. The detailed description of exosome generation is described in the methods (also in Suppl. Fig. 2).

Having confirmed by qPCR that exosomes loaded with scrambled mimic did not induce pro-inflammatory cytokines (TNFα, MCP1 and IL-1β) in target cells (Suppl. Fig. 1E and F), we next performed *in vivo* experiments. Intravenous administration of exo-miR-155 mimic (100 μl; ~2 × 10^8^ particles/ 100 μg exosomal protein) into miR-155 KO mice resulted in a ~15 fold increase in miR-155 levels in the plasma after 5 mins compared to naturally existing miR-155 plasma levels in wild type (WT) mice (Fig. 1C). By 30 mins, around 90% of miR-155 had disappeared from plasma and its levels in recipient miR-155 KO mice reached to the baseline miR-155 expression level of WT mice (Fig. 1C). This observation was specific to mice injected with exo-miR-155 mimic as no detectable levels of miR-155 was found in recipient miR-155 KO mice injected with scrambled mimic loaded exosomes (Fig. 1C–J). In perfused organs of recipient miR-155 KO mice, a peak in miR-155 levels was observed around 10 mins after exo-miR-155 mimic administration (Fig. 1C–H). Interestingly, we found that the kinetics of miR-155 was very similar in the perfused organs and plasma of recipient miR-155 KO mice (Fig. 1C–H). When compared to WT mice, the amount of miR-155 in recipient miR-155 KO mice was highest in the liver, followed by adipose tissue, lung, muscle, and lowest in the kidney. In the liver and adipose tissue of recipient miR-155 KO mice, the amount of miR-155 was higher at 10 mins compared to baseline expression of miR-155 in WT mice and returned to the baseline WT expression after 40 mins (Fig. 1D,E). A moderate increase in miR-155 levels was observed in the brain of recipient miR-155 KO mice after 10 mins, however it did not reach statistical significance due to sample variability (data not shown). No significant increase in miR-155 was observed in the thymus or heart.

To determine if exo-miR-155 mimic was delivered into cells, and not just to the interstitial space in the organs, we next evaluated miR-155 levels in isolated liver cell sub-populations. In hepatocytes and liver mononuclear cells (MNCs) of recipient miR-155 KO mice, a peak in miR-155 levels was found at 10 mins followed by a rapid decline in its levels similar to what we found in perfused liver/organs (Fig. 1I,J). No significant amount of miR-155 was found in spleen and bone marrow cells.

### Biological function of miRNA-155 mimic loaded exosomes in Kupffer cells isolated from miR-155 KO mice

**Figure 2:**
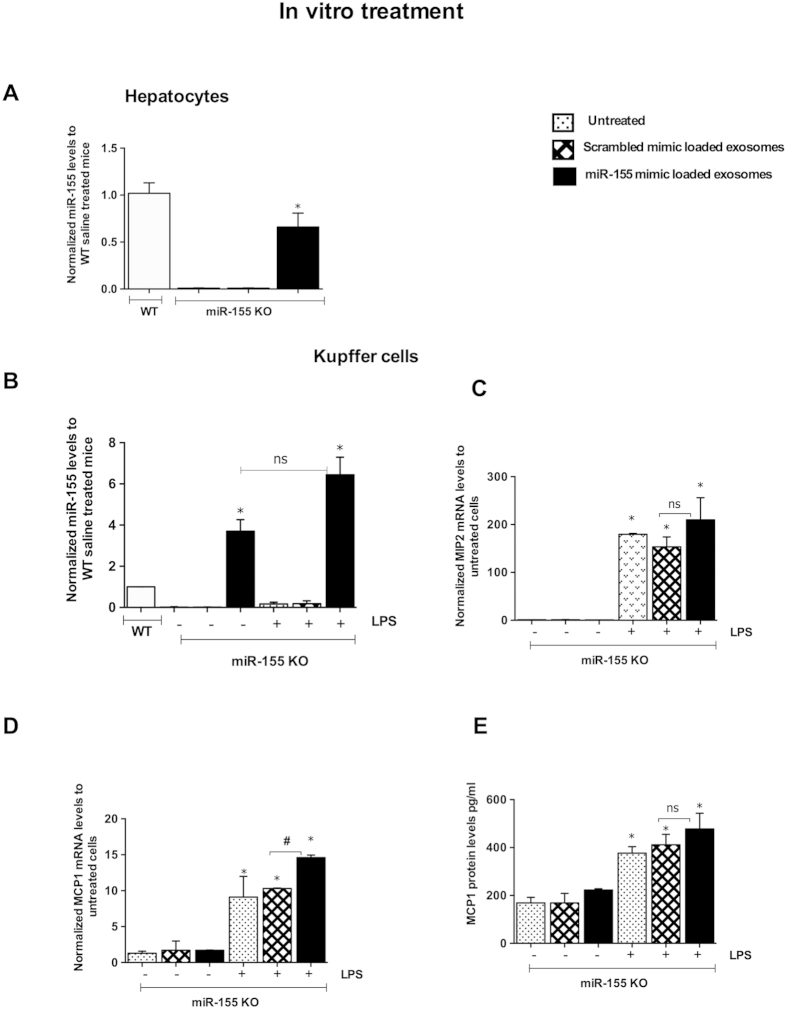
Biological function of exosome loaded miRNA-155 mimic. Hepatocytes and Kupffer cells were isolated from miR-155 KO mice as described in the methods. Exosomes loaded with either scrambled mimic or miR-155 mimic were added to cells for 6 h. Cells were washed 3 times with PBS to remove free-floating exosomes. Hepatocytes were lysed with Qiazole and miR-155 levels were evaluated (**A**) Kupffer cells were treated or not with LPS (100 ng/ul) for 6 h and cell-free supernatant was collected. The levels of miR-155 (**B**) MIP2 (**C**) and MCP1 (mRNA) were checked using real-time qPCR and levels were normalized to untreated cells. MCP1 protein levels were measured from cell-free supernatant (**E**) miR-155 levels were normalized to hepatocytes or Kupffer cells isolated from wild type mice. * indicates p<0.05 scrambled loaded exosome treated cells and # compared to scrambled loaded exosome +LPS treated cells. SnoRNA202 (**A**,**B**) or 18S (**C**,**D**) was used to normalize the technical variations between the samples. ns: non significant. Statistical analysis was performed using non-parametric Mann-Whitney test.

To evaluate the effect of exo-miR-155 mimic delivered to liver cells of miR-155 KO mice, we performed *in vitro* studies. *In vitro* co-culture of exo-miR-155 mimic with primary hepatocytes and Kupffer cells isolated from miR-155 KO mice resulted in detectable levels of miR-155 in both cell types (compared to cells treated with scrambled mimic exosomes) (Fig. 2A,B). Relative to WT miR-155 expression, introduction of exo-miR-155 mimic resulted in higher miR-155 expression in Kupffer cells than hepatocytes in miR-155 KO cells. (Fig. 2A,B). Also miR-155 levels in Kupffer cells isolated from miR-155 KO mice after co-culture with exo-miR-155 mimic were higher than the baseline miR-155 expression of WT cells (Fig. 2B). Functionally, hepatocytes treated with exo-miR-155 mimic showed no further increase in MCP1 protein production compared to those treated with scrambled mimic loaded exosomes after LPS challenge (data not shown). The restoration of miR-155 in Kupffer cells isolated from miR-155 KO mice resulted in the induction of pro-inflammatory cytokines after LPS challenge (Fig. 2C,E). Kupffer cells treated with exo-miR-155 mimic showed a higher induction in MIP2 mRNA (Fig. 2C) and MCP1 (both at the mRNA and protein levels) compared to cells received exosomes with scrambled mimic after LPS challenge (Fig. 2D,E). The scrambled or exo-miR-155 mimic did not induce any inflammatory response in LPS naïve cells (Fig. 2D,E).

### Biodistribution of wild type plasma in miR-155 KO mice

**Figure 3:**
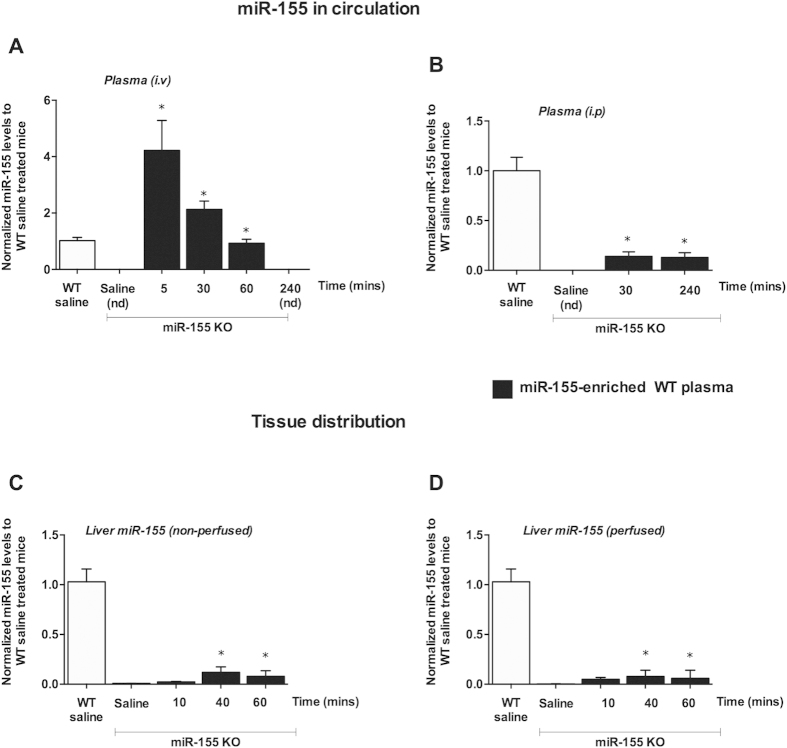
Biodistribution of transferred wild type plasma in miRNA-155 KO mice. miR-155 KO mice (n = 6–8) were injected with ~150 ul of plasma isolated from WT mice (CpG+LPS treated) as described in the methods. After systemic administration, blood was collected and mice were perfused or not as indicated. Plasma was isolated and miR-155 levels were determined by real-time qPCR. Due to miR-155 low signal, pre-amplification of cDNA from tissue samples was performed. i.v route of delivery (**A**) and i.p route of delivery (**B**) miR-155 levels in non-perfused (**C**) and perfused livers (**D**) miR-155 levels were normalized to wild type saline treated mice. Data is presented as mean ± SEM. * indicates p < 0.05 versus saline treated KO mice. nd: not detected. Synthetic spiked cel-miR-39 (**A**,**B**) or SnoRNA202 (**C**,**D**) was used to normalize the technical variations between the samples. Statistical analysis was performed using non-parametric Mann-Whitney test.

After observing a rapid biodistribution and organ and cellular uptake of exo-miR-155 mimic, we next evaluated the distribution of miR-155 present in WT plasma (natural). WT plasma was generated from CpG+LPS treated wild type mice and was enriched in miR-155 as we previously found^29^ (Fig. 1A). WT plasma was injected to miR-155 KO mice to determine the half-life and tissue distribution of natural miR-155. Intravenous administration of WT plasma (donor) (~150ul) into miR-155 KO (recipient) mice resulted in a ~ 4-fold increase in miR-155 levels in the plasma after 5 mins compared to WT mice (Fig. 3A). A peak in miR-155 levels was observed in the plasma of recipient miR-155 KO mice as early as 5 mins and there was a ~50% decrease in its levels after 30 mins of administration (Fig. 3A). After 1h, miR-155 levels in the plasma of recipient miR-155 KO mice were reduced to the baseline expression of WT mice. By 4 hours, miR-155 was undetectable in the plasma of recipient miR-155 KO mice. This data suggests that miR-155 from plasma of WT mice undergoes biodistribution in recipient miR-155 KO mice. In contrast to the rapid distribution of intravenous (i.v.) administered WT plasma, intraperitoneal (i.p.) route of delivery of plasma resulted in a lower but sustained levels of miR-155 in the plasma of recipient miR-155 KO mice even after 4h (Fig. 3B). For the remainder of the study we used i.v administration as the i.v route of plasma administration was more robust than i.p. to deliver miR-155.

Next, we evaluated whether reduction in plasma miR-155 levels after minutes post injection correlated with distribution of miR-155 into various tissues in recipient miR-155 KO mice. After i.v. administration of WT plasma, we found low but detectable levels of miR-155 in the liver of recipient miR-155 KO mice at 40 and 60 mins (Fig. 3C). After WT plasma transferred, signal of miR-155 in organs of recipient miR-155 KO mice was very low. Therefore, a pre-amplification of cDNA was performed to enhance miR-155 signals by real-time PCR. To rule out blood contamination accounting for miR-155 detection in tissues, we perfused the livers and red blood cell (RBC) lysis was performed using RBC lysis buffer. Similar to non-perfused livers, miR-155 was detected at 40 and 60 mins in perfused livers of recipient miR-155 KO mice (Fig. 3D), suggesting the accumulation of miR-155 in the liver rather than resulting from blood contamination. We also found very low levels of miR-155 in adipose tissue, and lung of recipient miR-155 KO mice (data not shown). No detectable level of miR-155 was found in kidney, thymus, bone marrow cells, heart, or brain of recipient miR-155 KO mice. This could be due to the fact that the injected amount of miR-155 was not enough to reach the detectable threshold in these organs of recipient miR-155 KO mice. Similarly, intraperitoneal administration of WT plasma resulted in a detectable but very low signal for miR-155 in the liver, and adipose tissue of recipient miR-155 KO mice (data not shown).

## Discussion

Circulating miRNAs have gained increasing interest as new biomarkers of various diseases. Aberrant miRNA signatures have been found in various diseases including cancers, metabolic and neurological disorders^7^,^33^. Circulating miRNAs can be packaged in extracellular vesicles including exosomes and microvesicles. Emerging studies suggest that extracellular miRNAs are involved in intercellular communication and exosome-mediated transfer of circulating miRNA has emerged as a new paradigm of horizontal transfer^6^,^34^,^35^. Here we demonstrated the biodistribution of highly studied inflammation associated miR-155.

**Figure 4:**
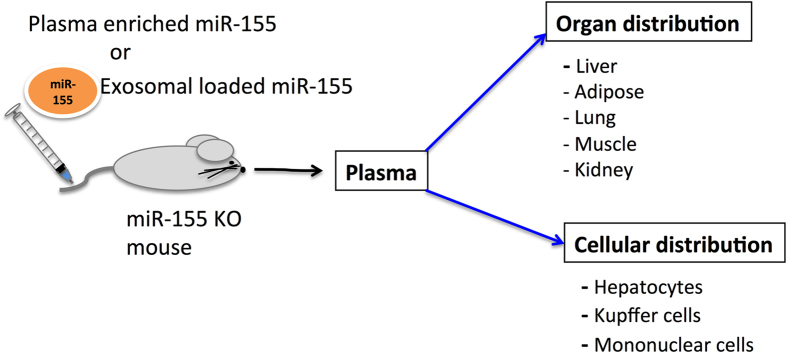
Summary of miR-155 biodistribution.

We report the distribution and half-life of exosome loaded miR-155 mimic (exo-miR-155 mimic) using the miR-155 KO mouse model system. We established a murine B cell derived exosome-mediated system to deliver a miR-155 mimic (mature) into mice. We demonstrated that exo-miR-155 mimic undergoes distribution in the recipient miR-155 KO mice very rapidly both in plasma and organs (Fig. 4). The half-life of exo-miR-155 mimic was short, around 5 mins. The highest amount of miR-155 was found in the liver followed by adipose tissue, lung and muscle and lowest in the kidney. Consistent with our results, previous studies have shown the tissue distribution of exogenously administered exosomes *in vivo* and fluorescent probe-labeled exosomes were predominantly detectable in the liver, lungs, kidneys, and spleen after 1h of intraperitoneal administration^36^,^37^. Our results indicate that exo-miR-155 mimic was delivered to hepatocytes and MNCs *in vivo* in the liver suggesting the potential use of exosomal-mediated delivery of miRNA/nucleic acids to different liver cell populations. In our study we found no significant accumulation of exo-miR-155 mimic in the brain. This is consistent with previous reports showing the delivery of exosomes into brain only after some modifications such as intranasal administration of the labeled exosomes^37^ and genetically modification of exosomes with a neuron-specific targeting peptide^25^. These studies suggest that modification either in exosomes or in delivery route is needed for successful delivery of exosomes into the brain. Our studies further demonstrate that exo-miR-155 mimic has a short half-life after systemic administration since most of exo-miR-155 mimic signal has disappeared from the organs and plasma after 30–40 mins post injection.

To rule out that anti-CD63 and/or anti-rabbit IgG immune complexes and/or magnetic beads do not account for the rapid clearance of exosomes *in vivo*, we carried out a pilot experiment where exosomes were isolated with ExoQuick only (before and after electroporation) and injected into mice for 30 mins. We found no differences in the kinetic of plasma levels of miR-155 in KO mice between isolation methods (anti-CD63 magnetic isolation versus ExoQuick). Consistent with our results, previous studies found that exosomes delivered to mice are rapidly cleared from the systemic circulation^38^,^39^,^40^,^41^,^42^. It was further demonstrated that hepatic and splenic macrophages take up exosomes administrated by intravenous injection. It was also suggested that the liver is involved in clearing of exosomes due to their negative charge using the same mechanism as in clearance of negatively charged liposomes^39^. These exosomes were prepared using ultracentrifugation method^38^,^39^. These studies including our pilot study rules out any possibility that rapid clearance of exosomes is due to anti-CD63/ or anti-IgG immune complexes or magnetic beads.

The advantage of exosome-mediated delivery of miR-155 mimic was that exosomes can be stored frozen (−80 ^°^C) for a prolonged time and are stable and ready to use without the need of immediate processing prior to administration. Also we found no inflammatory response in mice administered with scrambled mimic loaded exosomes suggesting the safe efficacy of exosomes for *in vivo* studies.

While electroporation of exosomes with miRNA or siRNA has been reported in the literature^25^, a more detailed study demonstrated that the majority of the siRNA precipitated outside exosomes and co-purified with exosomes^43^. We have used EDTA in our electroporation buffer and according to Kooijman *et al* there is less aggregation of miRNA in the presence of EDTA in electroporation buffer^43^. Moreover, RNAse A treatment performed after electroporation was able to degrade free floating miR-155 as we found difference in the amount of miR-155 in the exosomes before and after RNAse A treatment. If there are some miRNA aggregates attached to exosomes, it is likely that endogenous RNAse will degrade naked miRNA after administration to mice in our experimental conditions. There is no clear evidence if vesicular associated miRNA are either inside or just adhere to vesicles under *in vivo* conditions.

It has been demonstrated that chemically synthetic small RNA are instable in the serum^44^. miR-155 mimic used in our experiments has no chemical modifications such as LNA, o-methyl etc and naked miRNA is prone to degradation in the presence of RNAse and only miRNA associated with vesicles or proteins are resistant to RNAse digestion^2^,^5^. A recent study demonstrated that number of given miRNA in exosomes was less than one molecule^45^. These exosomes were isolated from normal human plasma, dendritic cells and ovarian cancer cells^45^. It is noteworthy to mention that in our experimental conditions we used higher amounts of miRNA mimic for electroporation into exosomes. The reason for using higher miRNA-155 amount was to have sufficiently levels to be able to detect in miR-155 KO mice after injection and follow its biodistribtuion.

Previously we showed induction of miR-155 in plasma (WT plasma) and circulating miR-155 was mostly fractionated with extracellular vesicles after CpG (TLR9 ligand) and LPS (TLR4 ligand) treatment in mice^29^. In the present study, we showed that delivery of WT plasma resulted in a rapid and robust accumulation of miR-155 in the plasma of recipient miR-155 KO mice. We provided evidence that injected miR-155 from WT plasma is distributed to the liver in recipient miR-155 KO mice. The half-life of donor plasma miR-155 in recipient miR-155 KO mice was around 30 mins. Our use of miR-155 KO mouse model system to study biodistribution of natural circulating miRNA/EV-associated miRNA could be applied to study distribution of other miRNA in future. Our results also indicated that the i.v route of plasma administration was more robust in comparison to the i.p mode of delivery. This observation was consistent with previous observation^46^. The differences in miR-155 levels with i.v. and i.p injections could also reflect miR-155 stability. However, there is not much known about the stability of circulating miRNA *in vivo*. One possibility could be that ip mode of administration releases miRNA slowly and accounts for the detection of miR-155 after 240 mins. Another possibility could be that vesicular associated miR-155 is retained more efficiently with i.p. than i.v. mode of administration. Vesicular associated miRNAs were shown to have increased stability^47^.

The advantage of using plasma miR-155 from wild type mice is that it contains miR-155 in both vesicles and non-vesicular forms^29^. The disadvantage is that CPG+LPS stimulation that facilitates miR-155 enrichment, also results in increased levels of cytokines/chemokines in the plasma^48^. Nevertheless, there is no evidence that carryover of cytokines would modulate the biodistribution of miR-155 in recipient miR-155 KO mice. Another limitation of using WT plasma was that the amount of miR-155 delivered to recipient miR-155 KO mice was very low. This limitation could be overcome by repeated injections (daily) of WT plasma over time to restore miR-155 to physiological levels to evaluate the biological role of WT circulating miR-155.

Biodistribution and dynamics of WT plasma were similar to that of exosome mediated delivery of miR-155, suggesting that both delivery modes have similar biodistribution profiles. However, we found the half-life of transferred plasma miR-155 was longer compared to exosome-loaded miR-155 in recipient miR-155 KO mice. This could be because exosomes are cleared faster in the liver^39^. Also WT plasma contains miR-155 both in EVs and non-vesicular forms^29^, which could account for increased stability of transferred plasma miR-155 *in vivo*.

Extracellular miRNAs have been reported to be involved in intercellular communication^11^,^12^,^13^. Indeed, exo-miR-155 mimic were readily taken up by primary mouse hepatocytes and Kupffer cells (KCs) isolated from miR-155 KO mice *in vitro* co-culture. Exosomal uptake was more efficient in Kupffer cells than hepatocytes. As highly proficient phagocytic cells, KCs are readily capable of uptake of microparticles^49^. This was in contrast to *in vivo* findings where relative miR-155 expression in hepatocytes was higher than MNCs isolated from recipient miR-155 KO mice. MNCs represented different liver population including monocytes, lymphocytes and dendritic cells and this could account for lower miR-155 expression *in vivo*. Of note: miR-155 basal expression is higher in KCs than hepatocytes in wild type mice^50^. The mechanisms of uptake of exosomes by cells likely varies with the cell type. In many pathways of uptake, exosomes will be trapped in the endosomes or lysosomes and eventually degraded^51^. Our results also indicate that miR-155 levels were different between total liver and isolated hepatocytes and MNCs *in vivo*. These differences could be attributed to different baseline expression of miR-155 in the wild type liver (Cq 33), isolated hepatocytes (Cq 32) and MNCs (Cq28). Another reason could be that the liver is composed of various cell population (Kupffer cells, dendritic cells, T cells, endothelial cells, NK, NKT) and it is likely that injected miR-155 is not only taken up by the hepatocytes and MNCs but also by other liver cells.

Our results indicate that miR-155 delivered by exosomes has functional effects in KCs as restoration of miR-155 in KCs isolated from miR-155 KO mice after co-culture with exo-miR-155 mimic resulted in pro-inflammatory gene induction after LPS treatment. We found only a moderate increase in these cytokines after introduction of exo-miR-155 mimics suggesting that KCs isolated from miR-155 KO mice had some defects in LPS signaling. This suggests that if exogenous miR-155 could be delivered in sufficient amounts it could be possible that KCs might take enough to have functional effect *in vivo*. Though our *in vitro* findings suggest that miR-155 loaded exosomes have a specific effect on MCP1, we cannot rule out the possibility that miR-155-electroporated exosomes could have multiple non-specific effects, including on innate immunity or by competition with miRNA, Ago proteins and other miRNA processing proteins in exosomes^52^.

In summary, the information in our study about the pharmacokinetics of plasma and exosome loaded miR-155 (Fig. 4) will help in developing exosome-mediated miRNA delivery and investigating the biological function *in vivo*. Future studies will be able to provide more information about other circulating miRNA distribution and function resulting in a better understanding of the role of these entities *in vivo*.

## Methods

All methods were carried out in accordance with the approved guidelines.

### Animal studies

Mice deficient in miRNA-155 (miR-155 KO) were obtained from Jackson Laboratory and the colony was maintained in the animal facility at the University of Massachusetts Medical School. Eight to ten week old C57/Bl6J wild type (WT) male or female mice were obtained from Jackson Laboratory. All experimental protocols were approved by the Institutional Animal Use and Care Committee of the University of Massachusetts Medical School (Worcester, MA). To induce the miR-155 induction in the circulation, wild type female mice were either injected with saline or 2.5 mg/kg CpG (i.p) for three days as described^53^. On day 4, CpG treated mice received 0.5 mg/kg LPS (i.p.) 3 h prior to sacrifice. At the end of treatment, mice were cheek bled and plasma was separated and stored at −80 ^°^C for further analyses. Liver and other tissue was washed with PBS and immediately either snap frozen in liquid nitrogen (protein analyses) or in RNA later (Qiagen) (RNA analyses) respectively.

To separate the plasma, blood was collected in EDTA containing microtainer tubes (BD Biosciences) and centrifuged at 5000 g for 10 mins at room temperature. The centrifugation step was repeated twice to minimize platelet contamination and the clear plasma fraction was aliquoted and stored at −80 ^0^C.

### Exosome isolation from tissue culture media

Exosomes were generated from the murine B cell line (M12.4.1). B cells were cultured in RPMI medium supplemented with 10% FBS and 1% penicillin/streptomycin. To induce exosome release, the cells were cultured in exosome-depleted FBS (System Biosciences, USA) medium stimulated with IL-4 (50 ng/mL)+CD40 (5 μg/mL) for 3 days as described^54^. IL-4 and CD40 treatment was used to increase exosome production as reported previously^54^.

For the isolation of exosomes, culture medium was centrifuged at 500 *g* for 10 mins to deplete cells and then at 10,000 *g* for 20 mins to eliminate residual cellular debris as described previously^22^. The resulting supernatant was passed through 0.4 μm and 0.22 μm filters and concentrated using the Amicon Ultra-15 Centrifugal Filter Unit with Ultracel-100 membrane (Millipore, MA). CD63 enriched exosomes were isolated using anti-CD63 immuno-magnetic positive selection as described^32^. Anti-CD63 antibody (Abcam cat. #ab8219 and Santa Cruz cat. #15363) was used as primary antibody followed by corresponding secondary antibody coupled with magnetic beads (Miltenyl biotech cat. #130-048-602). Exosomes were eluted from anti-CD63 beads using elution buffer from Invitrogen and finally exosomes were resuspended in PBS. The Miltenyl Biotec midi-MACS separator along side LS columns (cat. #130-042-901) was used.

The exosomes were quantified using NanoSight LM10 system (NanoSight, UK) equipped with a fast video capture and Nanoparticle Tracking Analysis system (NTA), according to the manufacturer’s instructions. The samples were measured for 60s at room temperature with manual shutter and gain adjustments. NTA was used to measure particle size (measured in nanometers) and concentration of particles (particles/ml). Each measurement was repeated for three times.

### Electron Microscopy

For electron microscopy, exosomes were re-suspended in PBS and placed on a formvar-coated copper grid and incubated for 30 mins as described^22^. The grid was washed with PBS and sample was fixed by placing the grid on the top of the 2% paraformaldehyde placed on the parafilm for 10 mins. Fixation was followed by several washes with deionized water and samples contrasted by adding 2% uranyl acetate for 15 mins. Samples were embedded by adding a drop of 0.13% methyl cellulose and 0.4% uranyl acetate for 10 mins. The grid was examined in a Philips CM10 transmission electron microscope and images were captured using a Gatan CCD digital camera.

### Western Blotting

Western blot analysis was carried out for exosomal and non-exosomal markers. Exosomes or B cells were lysed with RIPA buffer and 30 μg of protein from each fraction was run on 10% SDS-PAGE gel. Proteins were transferred to nitrocellulose membrane and were blocked in TBS containing 5% non-fat dry milk and 0.1% Tween-20 for 1 hour followed by incubation with primary CD63 or CD81 or GRP78 antibody at 4°C for overnight. Next day, membranes were washed 3 times with TBST and then incubated with corresponding horseradish peroxidase-conjugated secondary antibodies for 1 hour at room temperature (Santa Cruz Biotechnology). The target proteins were visualized on blot using a Clarity™ Western ECL substrate kit (BioRad) according to the manufacturer’s protocol and analyzed via Fujifilm LAS-4000 luminescent image analyzer.

### Loading of miR-155 mimic into exosomes

The loading of exosomes with miRNA-155 mimic or scrambled mimic was performed based on our previously optimized protocol^22^. Briefly, resuspended exosomes were diluted in the Gene Pulser electroporation buffer (Bio-Rad Laboratories, CA) in 1:1 ratio. Mouse miRNA-155 mimic (Ambion, NY) or scrambled miRNA mimic control#1 (scrambled mimic) at final concentration of 5 μmol/ml were added to 200 μl of exosome sample containing 1 μg/μl exosomal protein using standardized conditions as we previously described^22^. Approximately 1 μ mole of miRNA is loaded into 200 μl of exosome fractions, containing about 4 × 10^8^ particles (exosomes). The mixtures were transferred into cold 0.2cm electroporation cuvettes and electroporated at 150 V/100 μF capacitance using a Gene pulser II System (Bio-Rad Laboratories, CA). Immediately after electroporation, the mixture was treated with one unit of RNase A (Qiagen, Germany) for 30 mins to remove the free-floating miRNA mimic. Deactivation of RNase A was achieved by adding 2 μl of RNase inhibitor (Life technologies) and exosomes were re-isolated using ExoQuick-TC™ reagent as described by the manufacturer. The final pellet (exosome) was resuspended in PBS, aliquoted in 100 μl volume to avoid freeze and thaw cycles and stored at −80 °C. Of note, freeze and thaw of exosomes (2 times) did not influence miR-155 levels significantly.

### Delivery of WT plasma or miR-155 mimic loaded exosomes

Prior to injecting into recipient miR-155 KO mice, WT plasma (miR-155 enriched) or miR-155 mimic loaded exosomes (exo-miR-155 mimic) or scrambled loaded exosomes were brought to room temperature (thaw) and vortexed gently to make a homogenous suspension. 150 μl of WT plasma or 100 μl exosomes either containing miR-155 mimic or scrambled mimic was injected into recipient miR-155 KO mice (intraperitoneal [i.p.] or intravenous [i.v.]) for various times. Blood was drawn and animals were perfused using our laboratory standardized protocol^55^. To rule out any blood contamination red blood lysis buffer (Sigma, USA) was used as per manufacture’s instructions.

### Tissue collection, perfusion and cell isolation

At the conclusion of the experiment, various organs were removed, immediately washed in PBS, blotted on tissue and stored in RNA later at −80 ^°^C. To isolate the different liver cell populations, some mice were perfused using the laboratory protocol^55^. Briefly, mice were anesthetized with ketamine and the livers were perfused with saline solution for 10 mins followed by *in vivo* digestion with HBSS solution containing collagenase for 5 mins. The perfused liver was placed into petri dish containing HBSS and collagenase and cells were released by separating the liver lobes. To separate hepatocytes, cell suspension was centrifuged at 200 *g* for 5 mins at room temperature. The pellet containing hepatocytes was washed 2 times with PBS and cultured in low DMEM medium (10% FBS+antibiotics) containing insulin as described^55^. To isolate mononuclear cells (MNCs), supernatants were centrifuged at 1200 g for 10 mins and pellet was suspended in PBS and washed two times. Red blood lysis buffer (Sigma, USA) was used to remove blood contamination as per manufacture’s instructions. The resulting pellet was suspended in 40% percoll and layered on 70% percoll and centrifuged at 2000 g for 25 mins at room temperature. The interphase containing mononuclear cells were collected and washed with PBS 2 times and cultured in 10% low glucose DMEM containing antibiotics. Kupffer cells (KCs) were isolated as described previously using an established protocol^28^. Briefly, after 10 mins perfusion of liver with saline solution, *in vivo* digestion was done using liberase enzyme for 5 mins and followed by *in vitro* digestion for 30 mins. The non-hepatocyte fraction was separated by Percoll gradient and centrifuged at 1600 g for 30 mins. The inter-cushion layer was collected, washed with PBS two times and cultured in low glucose DMEM supplemented with 10% FBS and antibiotics. The non-adherent cells were removed after 3–4 h of plating with PBS and new medium was added. The KCs were used for subsequent experiments.

### *In vitro* co-culture of exosomes

To understand the biological effect of exo-miR-155 mimic, we performed *in vitro* studies. Primary hepatocytes, and Kupffer cells (KCs) were isolated from miR-155 KO mice and cultured with either control mimic or miR-155 mimic loaded exosomes (~2 × 10^8^ particles/ml) for 6 h. After incubation, cells were washed twice with PBS to remove free-floating exosomes. KCs were counted and plated onto 96 well plates (@1 × 10^6^ cells/ml) and stimulated with or without LPS (100 ng/ml) for 6 h. The supernatants were collected and centrifuged to remove the cell debris and used for MCP1 ELISA.

### RNA isolation and PCR analysis

10–20 mg of each organ was homogenized in QIAzol Lysis reagent (Qiagen, Germany) using stainless steel beads in TissueLyser II (Qiagen, USA). Total RNA was isolated using Direct-zol RNA MiniPrep kit with on column DNA digestion (Zymo Research Corp., USA). For mRNA analysis, cDNA synthesis was carried out using iScript reverse transcription system kit (BioRad, USA) and quantitative analyses of genes were performed using gene-specific primers on a Bio-Rad iCycler real time machine. Primer sequences for TNFα, MCP1, IL-1β and 18S were same as described^28^. Cq value was normalized to 18S and fold change was calculated using the delta-delta Ct method. For the detection of miRNAs, TaqMan miRNA Assays (Applied Biosystems) were employed as described previously^28^. SnoRNA202 (tissue/cells) or synthetic cel-miR-39 (exosomes and plasma) was used to normalize the technical variations between the samples. To enhance miR-155 signal in recipient miR-155 KO mice after the administration of WT plasma (miR-155 enriched) a pre-amplification step was introduced. The cDNA was pre-amplified using pre-amplification kit as described by the supplier (Applied Biosystem, USA) and diluted pre-amplified product was used for real time PCR.

### Enzyme-linked Immunosorbent Assay (ELISA)

Supernatants were collected from cultured KCs after co-culture and centrifuged at 2000 g for 10 mins to remove cellular debris and were frozen at −80 °C until use. Protein levels of TNFα, IL-1β and MCP1 were measured from cell-free culture supernatant by ELISA. The quantification of TNFα (BioLegends, San Diego, CA), MCP1 (BioLegend Inc., San Diego, CA) and IL-1β (R&D Systems, Inc., Minneapolis, MN) were carried out based on manufacturers’ recommendations using an ELISA reader.

### Statistical analysis

Statistical analysis was performed using non-parametric Mann-Whitney test. Data is shown as average ± standard error of the mean (SEM) and differences were considered statistically significant at p ≤ 0.05.

## Additional Information

**How to cite this article**: Bala, S. *et al.* Biodistribution and function of extracellular miRNA-155 in mice. *Sci. Rep.* **5**, 10721; doi: 10.1038/srep10721 (2015).

## Supplementary Material

Supporting Information
