# Supplementary material for: Biodistribution and function of extracellular miRNA-155 in mice
Source: Sci Rep. 2015 May 29;5:10721. doi: 10.1038/srep10721 (PMC4448655; doi:10.1038/srep10721)

Supplementary Information

Biodistribution and function of extracellular miRNA-155 in mice

Shashi Bala[1], Timea Csak[1], Fatemeh Momen-Heravi[1], Dora Lippai[3], Karen Kodys[1], Donna, Catalano[1], Abhishek Satishchandran[1], Victor Ambros[2] and Gyongyi Szabo[1]

University of Massachusetts Medical School, Department of Medicine[1], Department of Molecular Medicine[2], Worcester, MA 01605, Current address: 2nd Dept. of Internal Medicine, Semmelweis University, 1088-Budapest, Hungary[3]

Supplementary Figures

Supple Fig.1. Characterization of B cells derived exosomes. Exosomes were generated from murine B cells (M12.4) as described in the methods. Size distribution of exosomes was determined using Nanosight (A). Exosomes were visualized by transmission electron microscopy before and after electroporation (B). Equal amount of protein (30µg) from cells or exosome lysate was used for Western blot analysis using CD63, CD81 or GRP78 antibodies (C). Expression of miRNA-155 was measured in exosomes derived from naïve and IL-4+CD40 treated B cells. Spiked C.elegans miR-39 was used to normalize real-time PCR data (D). Liver mononuclear cells were isolated from WT mice and cultured with or without scrambled mimic loaded exosomes for 6h as described in the methods. Free-floating exosomes were removed and cells were cultured for another 6h. The mRNA levels of TNFα, MCP1 and IL-1β were determined from the cells using real-time PCR and 18S was used to normalize the technical variations between the samples (E). Cell-free supernant was used to measure protein levels of TNFα, MCP1 and IL-1β

by ELISA (F). Electroporation was used to load exosomes with miR-155 mimic. Loaded exosomes were either treated or not with 1unit of RNase A for 30 mins followed by inactivation by RNase inhibitor and re-isolation with ExoQuick TC as described in the methods. Total RNA was isolated and real-time qPCR was used to measure miR-155 levels. Synthetic spiked cel-miR-39 was used to normalize the technical variations between the samples (G). Statistical analysis was performed using non-parametric Mann-Whitney test.

Supple Fig.2. Flow diagram depicting exosome preparation from B cells and loading of miRNA-155 mimic into exosomes.

Supplementary Figure 1

A Size distribution of B cells exosomes

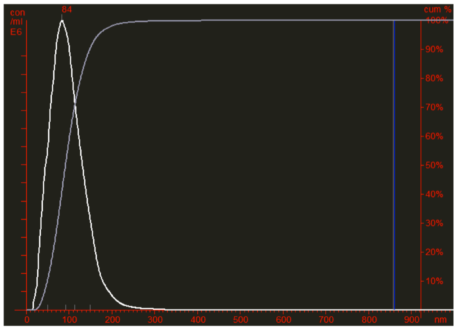

B TEM of B cells exosomes

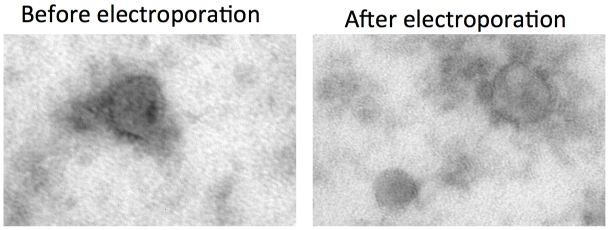

C

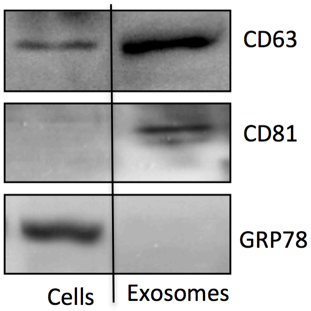

D

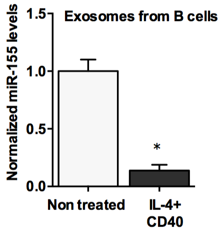

E

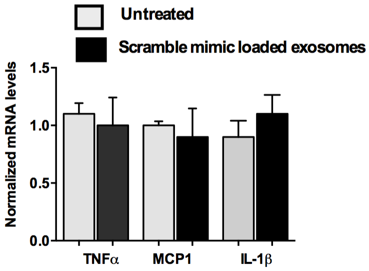

F

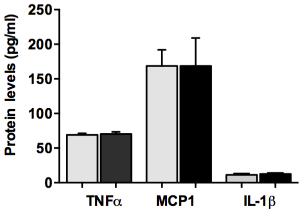

G

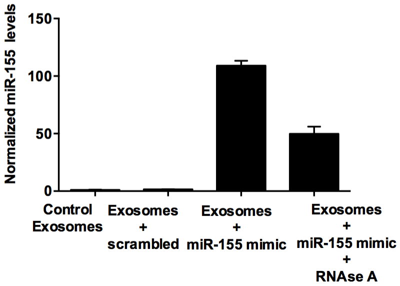

Supplementary Figure 2

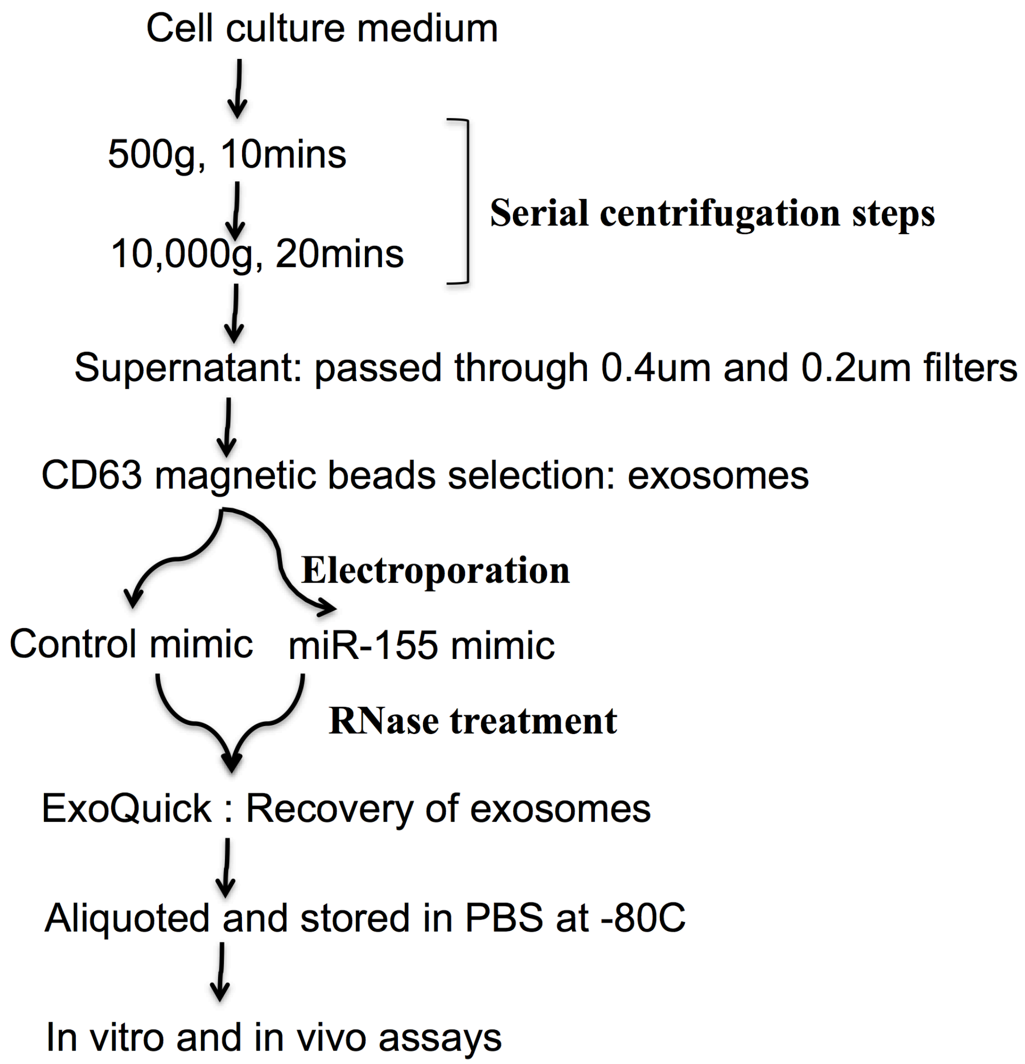

Supplement: Supporting Information [file srep10721-s1.pdf]
